# Eucain

**Published:** 1897-04

**Authors:** 


### Eucain.

One of the latest drugs which is now attracting attention is Eucain, and although several American and German periodicals have recently contained notes upon its uses, no English investigations have been published until the *British Medical Journal* recently printed a very instructive and useful paper upon its use as a local anæsthetic in the surgery of the throat, nose and ear. This article (which by-the-by, is only a "preliminary communication," based upon an experience of thirty-two cases) is the result of the joint investigations of Dr. Jobson Horne and Mr. Macleod Yearsley in their clinic at the Farringdon General Dispensary and Lying-in Charity. Their communication first deals with the history of the drug, giving a short summary of the work done up to date, both as to chemistry, pharmacology and therapeutics. Their own experiences are then detailed, and, unlike many modern investigators, they very wisely insist that, although they have so far formed a favorable opinion as to the merits of Eucain, it is but tentative, and they withhold their final verdict until they have still further examined into the uses of the drug. The points to which they appear to have especially directed attention are: 1, strength of solution required; 2, rapidity, intensity and extent of anæthesia; 3, general and local action upon the circulatory system; 4, after effects. Out of thirty-two cases they only met with three in wich there were any ill effects, and these seem to have had ample cause for not blaming the drug. Some of their results appear to be somewhat at variance with those of other observers; they have, for instance, noted slight ischemia rather than hyperemia of the turbinated bodies after its use. They also direct attention to a point which does not seem to have hitherto attracted attention, namely, the occurrence of salivation after its use. If the value of Eucain as a substitute for cocain for local anæsthesia be established, general practitioners will hail with delight the advent of a drug which, so far as the general result of present experience goes, is credited with all the advantages of cocain without its disadvantages.—*London Med. Times.*

